# Molecular Etiology and Mortality Risk Stratification of Non-SARS-CoV-2 Severe Acute Respiratory Infection in a Predominantly Pediatric Cohort: A Retrospective Study From a Tertiary Care Hospital in North India

**DOI:** 10.7759/cureus.116217

**Published:** 2026-09-14

**Authors:** Praroop Garg, Vikramjeet Dutta, Mohit Bhatia, Nawaz Ali, Rachna Sehgal, Pranav Ish, Amita Malik, Bindu Bajaj, Deepthi Nair

**Affiliations:** 1 Microbiology, Vardhman Mahavir Medical College and Safdarjung Hospital, New Delhi, IND; 2 Pediatrics, Vardhman Mahavir Medical College and Safdarjung Hospital, New Delhi, IND; 3 Pulmonary, Critical Care, and Sleep Medicine, Vardhman Mahavir Medical College and Safdarjung Hospital, New Delhi, IND; 4 Radiodiagnosis, Vardhman Mahavir Medical College and Safdarjung Hospital, New Delhi, IND; 5 Obstetrics and Gynecology, Vardhman Mahavir Medical College and Safdarjung Hospital, New Delhi, IND

**Keywords:** mortality, multiplex pcr, respiratory syncytial virus, sepsis, severe acute respiratory infection, vivi disease severity score

## Abstract

Background: Severe acute respiratory infections (SARI) remain a major cause of hospitalization and mortality, particularly among children and other vulnerable populations. Early identification of pathogens and disease severity is essential for clinical decision-making. This study evaluated the etiology of non-SARS-CoV-2 SARI and the prognostic utility of the ViVI Disease Severity Score (ViVI Score) in a predominantly pediatric cohort.

Methods: In this retrospective study, 117 hospitalized SARS-CoV-2-negative SARI patients, including 103 children and 14 adults, admitted to a tertiary care hospital in North India between September and December 2021 were evaluated. Respiratory pathogens were identified using multiplex polymerase chain reaction (PCR), alongside blood and lower respiratory tract cultures. Serum C-reactive protein (CRP), procalcitonin (PCT), and D-dimer were measured. Mortality prediction was assessed using the ViVI Score and receiver operating characteristic (ROC) curve analysis.

Results: The median age was 2.0 years (interquartile range, 0.5-7.5 years). Multiplex PCR detected respiratory pathogens in 69.2% of patients, with human respiratory syncytial virus (HRSV) A/B predominating. Blood cultures were positive in 40/117 patients (34.2%), predominantly yielding methicillin-sensitive *Staphylococcus aureus*. Overall mortality was 20.5%, increasing to 60% among patients with culture-confirmed sepsis. Elevated CRP and PCT were observed in all septic patients. The ViVI Score showed excellent mortality prediction (area under the curve (AUC) 0.946). A cutoff of ≥30 yielded 83.3% sensitivity and 91.4% specificity and was strongly associated with mortality (OR 53.1, 95% CI 14.5-194.0; p<0.001).

Conclusion: HRSV was the predominant respiratory pathogen, whereas bacterial sepsis was the principal determinant of mortality. Multiplex molecular diagnostics combined with standardized severity assessment may improve clinical triage, antimicrobial stewardship, and respiratory pathogen surveillance in resource-limited settings.

## Introduction

Severe acute respiratory infections (SARI) pose a significant global health challenge, causing substantial morbidity and mortality, particularly among vulnerable populations. The World Health Organization (WHO) defines SARI as an acute respiratory illness with a history of fever or measured fever of ≥38°C and cough, with onset within the last 10 days, requiring hospitalization [1]. However, this syndromic definition does not provide information regarding the underlying etiological agent or the individual risk of adverse outcomes. SARI may result from a wide range of viral and bacterial pathogens, and conventional diagnostic methods may not always provide a timely or comprehensive etiological diagnosis.

Before the COVID-19 pandemic, SARI detection was largely syndromic, relying on clinical presentation and limited diagnostic capacity [2]. The pandemic acted as a catalyst for strengthening surveillance systems and pathogen identification. In India, surveillance of epidemic-prone diseases is supported through the Integrated Disease Surveillance Programme, including laboratory-based and information technology-enabled surveillance [3].

SARI constitutes a major cause of healthcare utilization and hospitalization and may require referral to secondary and tertiary healthcare facilities, particularly among young children, older adults, and individuals with underlying comorbidities. Family physicians are often the first point of contact for patients presenting with acute respiratory symptoms and therefore play a pivotal role in early recognition of severe disease, timely referral, initiation of appropriate management, and implementation of infection prevention measures [1,2]. Accurate identification of respiratory pathogens and early risk stratification are essential to improve patient outcomes while minimizing unnecessary antibiotic use [2].

In low- and middle-income countries such as India, access to specialized diagnostic services remains limited at primary-care facilities, with sample transport and referral mechanisms used to improve access to diagnostic testing [4]. Consequently, evidence on the etiological profile of SARI and validated clinical severity assessment tools is highly relevant for strengthening referral pathways, antimicrobial stewardship, and respiratory disease surveillance. Such evidence can assist family physicians and primary care providers in identifying patients requiring urgent referral, promoting rational antimicrobial use, and optimizing utilization of healthcare resources [2,4].

To standardize clinical assessment, various severity scoring systems have been introduced. One of the most widely recognized is the ViVI Disease Severity Score (ViVI Score), developed by the Vienna Vaccine Safety Initiative. This 22-item composite score quantifies the clinical severity of influenza-like illnesses and SARI across diverse patient populations [5]. It includes indicators of both uncomplicated and complicated disease, providing a standardized framework for clinical decision-making, disease severity assessment, and epidemiological comparison.

The advent of multiplex polymerase chain reaction (PCR) platforms has markedly advanced the diagnostic approach to SARI by enabling simultaneous detection of multiple respiratory pathogens from a single clinical specimen, thereby improving diagnostic accuracy and expediting clinical decision-making [6]. Integration of rapid molecular diagnostics with standardized severity assessment has the potential to improve clinical risk stratification while strengthening pathogen-specific surveillance and facilitating the integration of virological and clinical data for surveillance and public health applications [6,7].

Despite advances in molecular diagnostics and standardized severity assessment, evidence integrating pathogen identification with clinical risk stratification in non-SARS-CoV-2 SARI remains limited, particularly from resource-constrained settings. Addressing this gap may help strengthen clinical decision-making, antimicrobial stewardship, and respiratory infection surveillance across primary and tertiary healthcare settings.

## Materials and methods

Study design and setting

This retrospective observational study analyzed laboratory and clinical data of patients admitted with severe acute respiratory syndrome during the defined period from September to December 2021, corresponding to the COVID-19 pandemic, at a tertiary care teaching hospital in North India. The data analysis was undertaken subsequently in the post-pandemic phase. The study was approved by the Institutional Ethics Committee of Vardhman Mahavir Medical College and Safdarjung Hospital, New Delhi, India (Approval Ref No. IEC/VMMC/SJH/Cert./05-2024/54, dated 23/07/2024).

The primary objective was to establish an etiological profile of pathogens responsible for SARI other than SARS-CoV-2 among hospitalized patients, using a combination of multiplex PCR and conventional microbiological culture. Clinical and laboratory data were retrieved retrospectively from hospital records. The study population was predominantly pediatric, comprising 103 children and 14 adults.

Sampling technique and patient selection

Consecutive patients admitted during the study period with signs and symptoms of acute respiratory illness were screened for inclusion. Patients meeting the WHO SARI case definition, with fever ≥38°C and cough, with symptom onset within the last 10 days, requiring hospitalization, were included in the study [1]. The study cohort comprised predominantly pediatric patients, with 103 children and 14 adults.

Pathogen detection and laboratory methods

RT-PCR for SARS-CoV-2

Nasopharyngeal and/or oropharyngeal swabs were collected from all SARI patients in the hospital during that period and tested for SARS-CoV-2 RT-PCR. Patients who tested negative for SARS-CoV-2 were included in this study and were subjected to multiplex PCR. Testing was performed in a designated COVID-19 molecular diagnostic laboratory, following Indian Council of Medical Research (ICMR) guidelines [8] and using ICMR-National Institute of Virology-approved commercial RT-PCR kits. The test targeted specific viral genes (e.g., E, ORF) for detection of SARS-CoV-2 RNA.

Multiplex PCR for Respiratory Pathogens

Respiratory specimens (nasopharyngeal/throat swabs) were further subjected to multiplex PCR using the Fast-Track Diagnostics Respiratory Pathogens 21 Plus (Fast-Track Diagnostics, Junglinster, Luxembourg). This assay detects *Chlamydia pneumoniae*, Enterovirus, *Haemophilus influenzae B*, Human adenovirus, Human bocavirus, Human coronavirus 229E, Human coronavirus HKU1, Human coronavirus NL63, Human coronavirus OC43, Human metapneumoviruses A/B, Human parainfluenza virus 1-4, Human respiratory syncytial virus (HRSV) A/B, Human rhinovirus, Influenza A virus, Influenza A (H1N1) virus, Influenza B virus, *Mycoplasma pneumoniae, Staphylococcus aureus, *and* Streptococcus pneumoniae.* Tests were conducted according to the manufacturer's protocol, including appropriate internal and external controls.

Conventional Microbiological Culture

Blood cultures were performed in all 117 enrolled patients using the BacT/ALERT® automated system (bioMérieux, France). Samples that flagged positive were subcultured on blood and MacConkey agar plates, followed by identification using standard microbiological techniques [9,10]. Patients with positive blood cultures were clinically evaluated and classified as having sepsis based on the treating clinical team's assessment and documented clinical findings. All 40 patients with positive blood cultures were clinically diagnosed with sepsis. 

The lower respiratory tract samples were processed using standard microbiological techniques [9,10]. Isolates were identified by morphology, Gram staining, and biochemical profiling.

Biomarkers

Serum C-reactive protein (CRP), procalcitonin (PCT), and D-dimer were measured on the Mispa i3 automated cartridge-based specific protein analyser (Agappe Diagnostics Ltd., Kerala, India). The platform uses a dual-channel system (rate-based nephelometry and turbidimetry/photometry) and prefilled single-use reagent cartridges with test-specific IC-card (smart card) calibration.

Disease severity assessment

The ViVI Score was used to assess disease severity based on 22 clinical parameters, with a total score ranging from 0 to 48 [11,12]. The scoring was weighted as follows: Disease Severity with Uncomplicated Symptoms (DSU): Weight=1×; Disease Severity with Complicated Symptoms (DSC): Weight=3×; Total Disease Severity Score=DSU + DSC. A schematic representation of the study methodology is shown in Figure 1.

**Figure 1:**
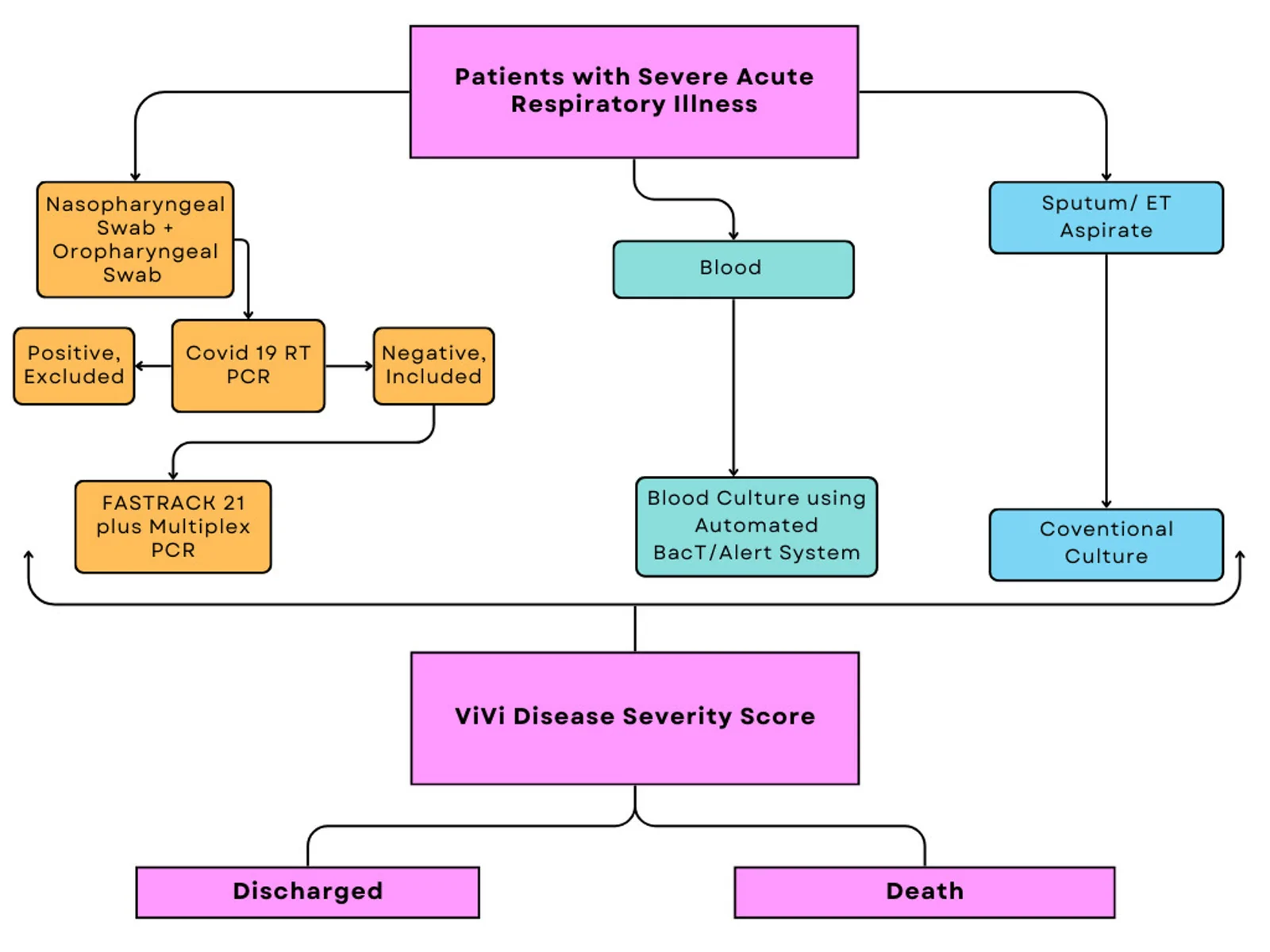
Schematic representation of the study methodology Fast-Track 21 (Fast-Track Diagnostics, Junglinster, Luxembourg); BacT/ALERT® (bioMérieux, France) PCR: polymerase chain reaction; ET: endotracheal

Statistical analysis

Pathogen distributions and descriptive statistics (median, IQR) were calculated in Microsoft Excel (Microsoft Corporation, Redmond, Washington, United States), and odds ratios were estimated using Fisher’s exact test. Diagnostic performance of the ViVI Score for outcome classification was assessed using receiver operating characteristic (ROC) analysis of ordinal rating data in JROCFIT v2.0.1 (John Eng; based on ROCFIT by Metz et al.) [13]: outcomes were coded as positive = survival (alive) and negative = death, ViVI scores were converted into an ordinal rating scale in which higher categories represented stronger evidence of the positive state, and a maximum-likelihood unequal-variance binormal ROC model was fitted to estimate the area under the curve (AUC) with its standard deviation; because ViVI is a severity index (higher scores indicate poorer prognosis), the ratings were oriented such that lower ViVI scores corresponded to stronger evidence of survival.

## Results

A total of 117 patients were included in the study. The cohort was predominantly pediatric, comprising 103 children and 14 adults. The median age of the study population was 2.0 years (IQR 0.5-7.5 years). The male-to-female ratio was 1.81:1, indicating a male predominance.

Pathogen detection profile from the multiplex PCR

Out of the 117 patients, 81 (69.2%) were positive for at least one respiratory pathogen using multiplex PCR. Multiplex respiratory pathogen testing identified HRSV A/B as the most frequently detected pathogen (n=60), followed by *Streptococcus pneumoniae* (n=16), *Staphylococcus aureus* (n=11), human rhinovirus (n=8), and influenza B virus (n=7). Lower detection rates were observed for human parainfluenza virus type 4 (n=3), human adenovirus (n=2), human coronavirus OC43, human metapneumovirus A/B, human parainfluenza virus type 3, and human parechovirus (each n=1). No cases were detected for *Chlamydophila pneumoniae*, enterovirus, *Haemophilus influenzae*, human bocavirus, human coronaviruses 229E, HKU1, and NL63, human parainfluenza virus types 1 and 2, influenza A virus (including H1N1), or *Mycoplasma pneumoniae*. Conversely, 36 patients tested negative for all pathogens, indicating either non-infectious etiologies or undetected organisms outside the panel’s scope (Figure 2). Additionally, six cases of acute respiratory distress syndrome (ARDS) due to HRSV were documented in a subset of adult patients, along with HRSV positivity among 13 children with underlying hematological disorders.

**Figure 2:**
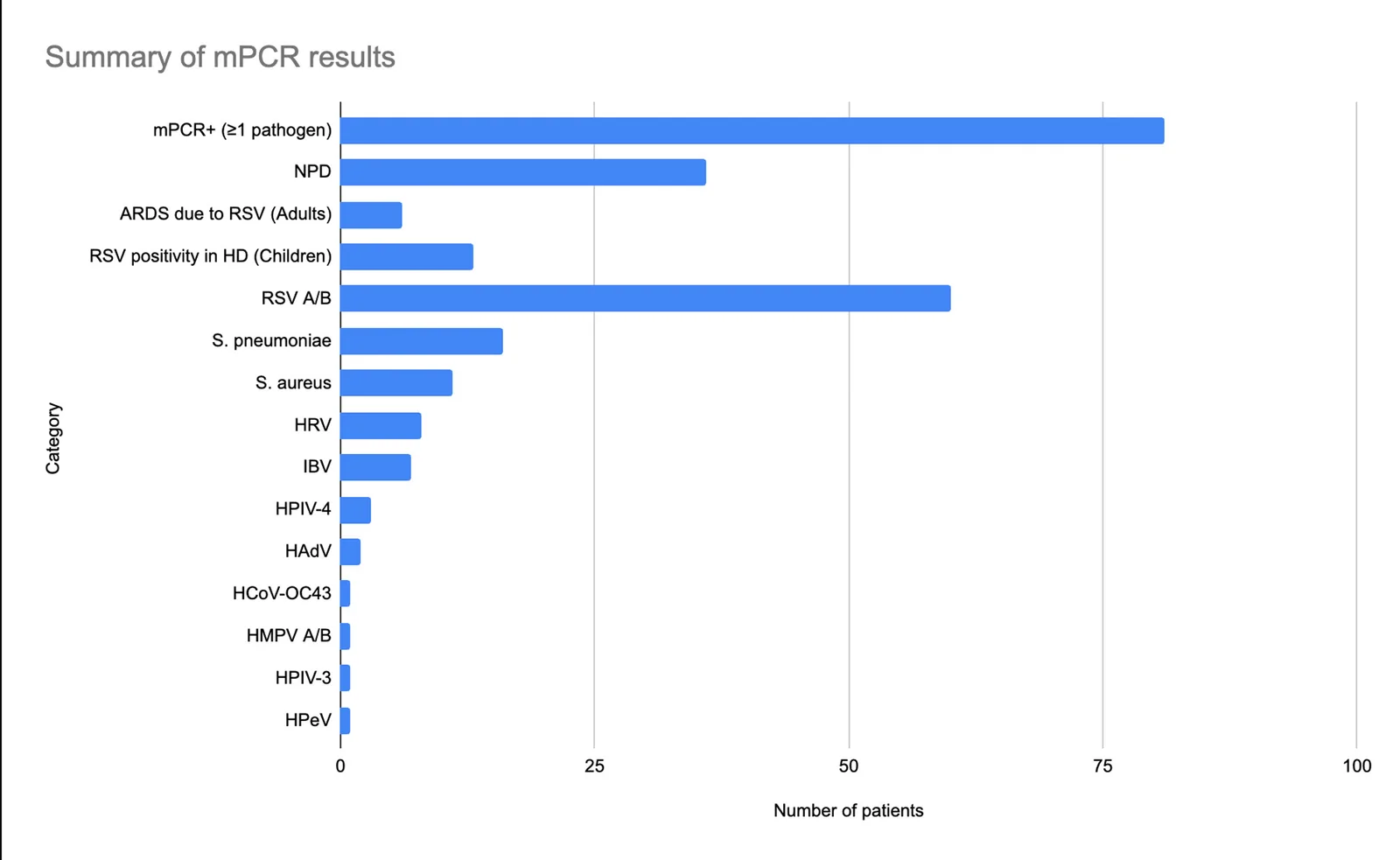
Distribution of pathogen positivity by multiplex PCR in patient samples HRSV A/B was the most frequently identified organism, followed by *Streptococcus pneumoniae, Staphylococcus aureus*, and Rhinovirus. mPCR+: multiplex polymerase chain reaction positive for at least one pathogen; RSV A/B: respiratory syncytial virus types A and B; *S. pneumonia: Streptococcus pneumoniae*; *S. aureus: Staphylococcus aureus*; HRV: human rhinovirus; IBV: influenza B virus; HPIV-4: human parainfluenza virus type 4; HAdV: human adenovirus; HCoV-OC43: human coronavirus OC43; HMPV A/B: human metapneumovirus types A and B; HPIV-3: human parainfluenza virus type 3; HPeV: human parechovirus; NPD: no pathogen detected; ARDS: acute respiratory distress syndrome; HD: hematological disorders

Pathogen detection profile from conventional cultures

Blood cultures were positive in 40/117 patients (34.2%); five were adults, and 35 were children. Among the positive blood cultures, 18 yielded methicillin-sensitive *Staphylococcus aureus*, seven *Escherichia coli*, seven *Klebsiella pneumoniae*, seven *Streptococcus pneumoniae*, and one yielded both *S. aureus *and *S. pneumoniae*. Lower respiratory tract aerobic cultures were positive in 48 patients (five adults and 43 children), yielding *S. pneumoniae* in 15, *E. coli* in 12, confluent growth of *K. pneumoniae* in 10, and *S. aureus* in 10; one patient had both *S. pneumoniae* and *S. aureus*.

Biomarker profile

Elevated PCT (>0.5 ng/mL) was observed in 55/117 patients (47.0%), including all 40 patients with positive blood cultures. Markedly elevated CRP (>10 mg/dL) was observed in 101/117 patients (86.3%), including all 40 patients with positive blood cultures. Positive D-dimer, suggestive of a hypercoagulable state, was observed in 35 patients, of whom 30 had sepsis.

ViVI Score distribution

The median ViVI Score across all patients was 22. Distribution of ViVI scores varied based on pathogen and infection type. Among single infections, HRSV A/B was most frequent (n=38) with a median ViVI score of 20.5 (IQR 13.5). Other single pathogens were less common, including *Staphylococcus aureus *(n=5; median 20 (IQR 19)), Influenza B (n=4; median 20.5 (IQR 10.25)), *Streptococcus pneumoniae* (n=3; median 29 (IQR 6.5)), Human rhinovirus (n=2; median 35 (IQR 6)), and individual cases of adenovirus (median 37) and parainfluenza virus 4 (median 42). Among coinfections, HRSV + *Streptococcus pneumoniae* was the most common (n=10; median 21 (IQR 13.75)). Other HRSV coinfections included HRSV + rhinovirus (n=3; median 20 (IQR 4)), HRSV + Influenza B (n=2; median 32 (IQR 16)), and HRSV + *Staphylococcus aureus* (n=2; median 20 (IQR 3)), while the remaining coinfections occurred as single cases with variable ViVI scores (Table 1).

**Table 1:** Distribution of median ViVI Scores across respiratory pathogens and coinfections The table summarizes the median ViVI score with the corresponding interquartile range (IQR) for each identified organism or organism combination, along with the number of affected patients and classification as single infection or coinfection. ViVI scores reflect clinical severity at presentation, with higher scores indicating greater disease severity. Coinfections include viral-viral and viral-bacterial combinations detected by multiplex PCR and/or standard microbiological methods. HRSV: human respiratory syncytial virus; PCR: polymerase chain reaction; ViVI Score: ViVI Disease Severity Score

Organism / Coinfection	Median ViVI score	IQR (interquartile range)	Number of patients	Type of infection
Human respiratory syncytial viruses A/B	20.5	13.5	38	Single Infection
Staphylococcus aureus	20	19	5	Single Infection
Influenza B virus	20.5	10.25	4	Single Infection
Streptococcus pneumoniae	29	6.5	3	Single Infection
Human rhinovirus	35	6	2	Single Infection
Human adenovirus	37	0	1	Single Infection
Human parainfluenza virus 4	42	0	1	Single Infection
HRSV + *Streptococcus pneumoniae*	21	13.75	10	Coinfection
HRSV + Human rhinovirus	20	4	3	Coinfection
HRSV + Influenza B virus	32	16	2	Coinfection
HRSV + *Staphylococcus aureus*	20	3	2	Coinfection
Adenovirus + HRSV	16	0	1	Coinfection
Coronavirus OC43 + HRSV	16	0	1	Coinfection
Metapneumovirus + HRSV	45	0	1	Coinfection
Parainfluenza virus 3 + Parainfluenza virus 4	11	0	1	Coinfection
Parainfluenza virus 4 + Parechovirus	8	0	1	Coinfection
Rhinovirus + *Staphylococcus aureus*	29	0	1	Coinfection
Influenza B virus + *Streptococcus pneumoniae*	29	0	1	Coinfection
Staphylococcus aureus + *Streptococcus pneumoniae*	35	0	1	Coinfection
HRSV + Parainfluenza virus 4	15	0	1	Coinfection
HRSV + Influenza B + *Staphylococcus aureus*	27	0	1	Coinfection

Clinical outcomes and mortality

The overall mortality rate in the cohort was 20.5% (24/117). Of particular concern was the subgroup of patients presenting with SARI and culture-confirmed sepsis (n=40), in which mortality rose dramatically to 60% (24/40). This suggests that progression to sepsis remains the strongest indicator of poor outcomes in SARI cases.

Predictive utility of the ViVI Score

A total of 117 cases were analyzed (93 survivors and 24 deaths). ROC analysis using JROCFIT demonstrated excellent discrimination between survivors and non-survivors (fitted AUC 0.946 (SD 0.022); empiric AUC 0.942) (Figure 3).

**Figure 3:**
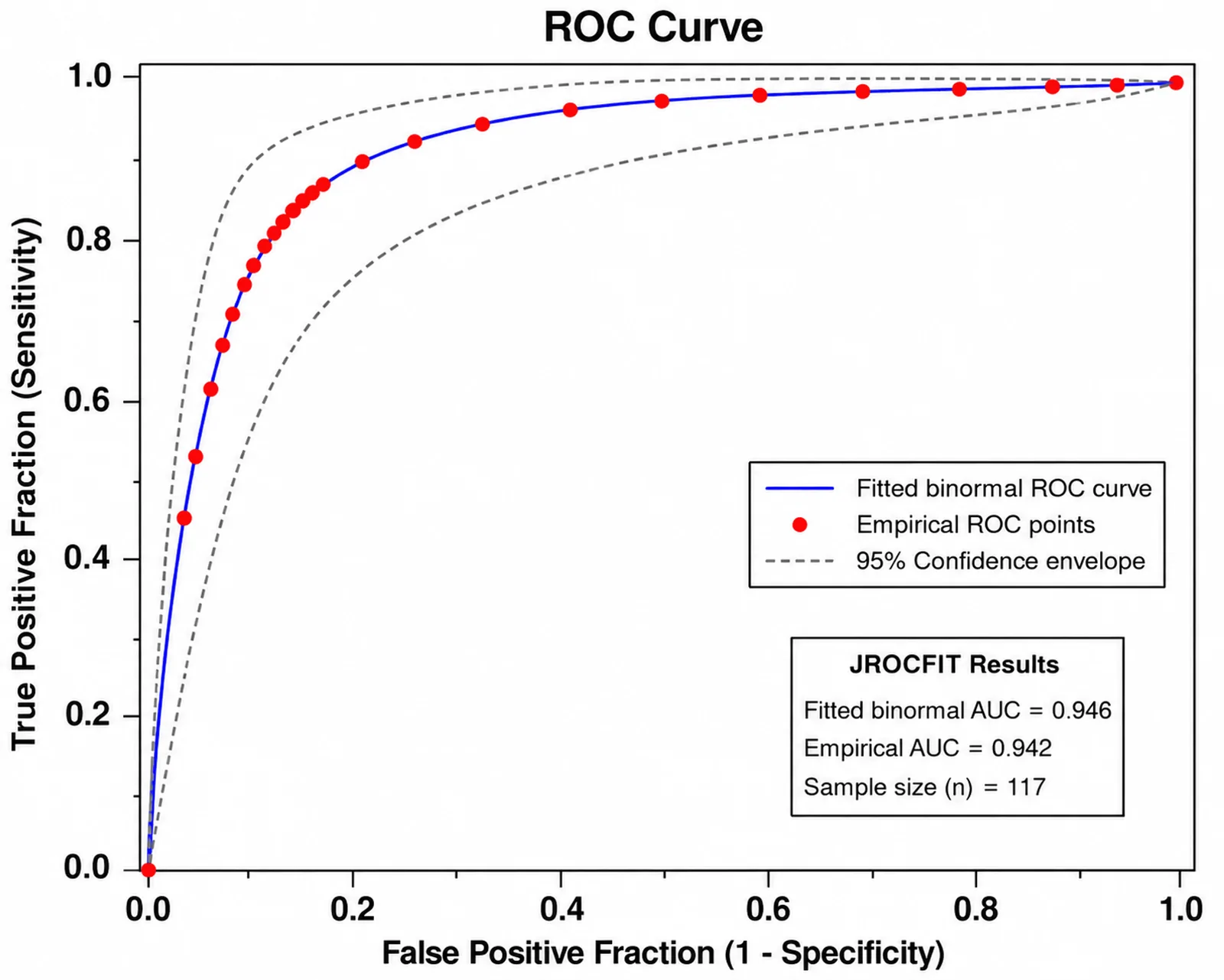
Receiver operating characteristic (ROC) curve demonstrating the predictive performance of the ViVI Disease Severity Score for mortality The ROC plot displays the relationship between the false positive fraction (FPF: 1 − specificity) on the x-axis and the true positive fraction (TPF: sensitivity) on the y-axis across decision thresholds. The blue curve with red markers represents the binormal ROC curve fitted using maximum-likelihood estimation (JROCFIT), while the grey envelopes depict the 95% confidence interval around the fitted curve. The empirical (nonparametric) ROC operating points are shown as black markers (with the green connection, where applicable). Overall discrimination was excellent (fitted AUC ≈ 0.946; empirical AUC ≈ 0.942), with the curve lying close to the upper-left corner, indicating high sensitivity at low false-positive rates.

The ROC curve lies close to the upper-left corner, indicating excellent discriminatory performance of the ViVI Score for predicting mortality across a range of decision thresholds (Figure 3). Using the ViVI Score as a mortality predictor and classifying death as the event, a low cutoff of ViVI ≥15 yielded 100% sensitivity for death (24/24) but low specificity (29.0%, 27/93) (TP=24, FN=0, TN=27, FP=66). In contrast, a high cutoff of ViVI ≥45 yielded 100% specificity (93/93) but low sensitivity (25.0%, 6/24) (TP=6, FN=18, TN=93, FP=0). The optimal threshold by Youden's J was ViVI ≥30, which provided balanced performance (sensitivity 83.3% (20/24) and specificity 91.4% (85/93); overall accuracy 89.7% (105/117)) (TP=20, FN=4, TN=85, FP=8). Patients with ViVI ≥30 had markedly increased odds of death compared with those with ViVI <30 (odds ratio 53.1, 95% CI 14.5-194.0; Fisher's exact test p=1.4 × 10⁻¹²).

## Discussion

The present study provides a comprehensive etiological and clinical profile of non-SARS-CoV-2 SARI in a predominantly pediatric cohort, with simultaneous assessment of respiratory pathogens, bacterial culture results, inflammatory biomarkers, and disease severity. Multiplex PCR detected at least one respiratory pathogen in 69.2% of patients, while HRSV A/B was the predominant respiratory pathogen. Culture-confirmed bloodstream infection was associated with substantially higher mortality, and the ViVI Score demonstrated excellent discrimination for mortality.

The 69.2% pathogen detection rate observed with multiplex PCR is consistent with previous studies demonstrating substantial pathogen detection among patients with severe respiratory infection. El Kholy et al. reported a high rate of viral detection and frequent co-detection in children with SARI, while Contier et al. demonstrated the diagnostic performance of multiplex PCR among critically ill immunocompromised patients [14,15]. Differences in age distribution, disease severity, sampling practices, seasonality, and the respiratory pathogen panels used may account for variations in diagnostic yield between studies.

HRSV A/B was the predominant respiratory pathogen in our cohort. This finding is consistent with surveillance studies from different geographical settings, including Senegal, India, and Suriname, which have identified HRSV as an important cause of severe respiratory illness and hospitalization among children [16-18]. The global burden of HRSV is substantial. A systematic analysis estimated 33.0 million HRSV-associated acute lower respiratory infection episodes, 3.6 million hospital admissions, and 101,400 deaths among children younger than five years in 2019, with the majority of deaths occurring in low- and middle-income countries [19].

The frequency of co-infections observed in our cohort is also consistent with previous pediatric SARI studies using multiplex molecular platforms. Lamrani Hanchi et al. reported pathogen detection in 72.5% of hospitalized children and co-infection in 23.3%, highlighting the ability of multiplex PCR to identify multiple respiratory pathogens from a single clinical specimen [20]. Similarly, studies incorporating digital surveillance and virological characterization have emphasized the value of combining pathogen detection with standardized clinical information [21,22]. These findings support the use of syndromic molecular testing as an adjunct to conventional microbiological investigations in patients with severe respiratory disease. However, detection of a respiratory pathogen does not necessarily establish causality, particularly for respiratory viruses that may be detected during prolonged shedding or asymptomatic infection. Therefore, multiplex PCR results should be interpreted in conjunction with clinical findings, disease severity, and conventional microbiological results.

The exceptionally high mortality observed among SARI patients with culture-confirmed sepsis (60%) was substantially higher than the overall mortality in the cohort. This finding is consistent with pediatric intensive-care data demonstrating significant mortality among children with bloodstream-infection-associated severe sepsis [23]. The finding emphasizes the importance of early recognition of bacterial superinfection and sepsis among patients presenting with severe respiratory illness, particularly when clinical deterioration or evidence of systemic infection is present.

HRSV-associated disease severity is influenced by age and underlying clinical conditions. Recent multicenter pediatric data have demonstrated increased risk of severe RSV disease among younger children, premature infants, and older children with pulmonary or neurological, neuromuscular, or developmental conditions [24]. In our cohort, children with underlying hematological disorders represented an important high-risk subgroup, and HRSV-associated ARDS was associated with severe clinical outcomes. These findings reinforce the importance of identifying underlying risk factors when assessing patients with HRSV-associated SARI.

The ViVI Score provided a structured approach to clinical severity assessment. Previous work has emphasized the importance of standardized severity assessment for respiratory viral illness and SARI [5]. More recent severity scales have also been evaluated to support respiratory hospitalization surveillance and viral genomic surveillance [25]. In our cohort, a ViVI score of ≥30 demonstrated excellent discrimination for mortality, with an AUC of 0.946. This finding supports the potential utility of standardized severity assessment for identifying patients at increased risk of poor outcomes. However, prospective validation in independent populations is required before this threshold can be considered for broader clinical implementation.

Raised PCT and CRP concentrations were associated with culture-positive sepsis and adverse clinical outcomes in our cohort. Previous pediatric data have evaluated PCT and CRP as markers of sepsis, with PCT potentially providing useful information regarding bacterial infection and disease severity [26]. However, neither biomarker should be interpreted as a stand-alone diagnostic test. Their value is greatest when interpreted together with the clinical presentation, microbiological findings, and other laboratory parameters.

The findings also have implications for respiratory disease surveillance and clinical triage. Integration of rapid molecular diagnostics with standardized severity assessment can provide information on both pathogen circulation and clinical severity [21,22,25]. Such an approach may be particularly valuable in resource-constrained settings, where molecular diagnostic capacity is concentrated at tertiary-care facilities, and timely referral of patients with severe disease remains important. In primary-care settings, recognition of clinical warning signs and timely referral remain essential, while tertiary-care molecular testing can provide additional etiological information.

The post-pandemic period is particularly relevant for respiratory pathogen surveillance because changes in population immunity, non-pharmaceutical interventions, and patterns of viral circulation may influence the timing and intensity of respiratory virus activity [16]. Continued surveillance is therefore important for identifying changing epidemiological patterns, detecting populations at increased risk of severe disease, and informing healthcare preparedness.

The predominance of HRSV in our cohort also highlights the importance of preventive strategies. Current approaches to prevention of severe RSV disease include maternal vaccination and long-acting monoclonal antibody strategies, particularly for infants at greatest risk of severe disease [27]. Continued pathogen-specific surveillance will be important for evaluating the epidemiological impact of these interventions.

The study has several strengths. It integrates multiplex molecular diagnostics with conventional microbiological culture, inflammatory biomarkers, and standardized severity assessment, allowing simultaneous evaluation of pathogen distribution, bacterial superinfection, disease severity, and mortality. The use of a standardized severity score also enabled quantitative assessment of the relationship between clinical severity and mortality.

In this study, several limitations should nevertheless be considered. The retrospective single-center design and relatively small sample size limit generalizability. The cohort was predominantly pediatric (103 children and 14 adults), which reflects the population evaluated at our center during the study period. Consequently, the findings, particularly those related to pathogen distribution, disease severity, and mortality risk stratification, should be interpreted primarily in the context of pediatric SARI and may not be directly generalizable to adult SARI populations. The multiplex PCR panel had a finite pathogen spectrum and may not have identified organisms outside the panel. Molecular detection cannot always distinguish active infection from asymptomatic carriage or prolonged nucleic acid detection. In addition, the ViVI mortality threshold identified in this cohort requires prospective external validation before it can be generalized to other populations or used to guide clinical management.

## Conclusions

HRSV was the predominant respiratory pathogen identified among hospitalized patients with non-SARS-CoV-2 SARI. Multiplex molecular testing provided useful etiological information, while the ViVI Score demonstrated good discriminatory ability for mortality risk in this cohort. Further prospective studies in larger and more diverse populations are needed to validate these findings.
